# Analysis of COVID-19 reinfection and its influencing factors among primary healthcare workers in Jiangsu Province: a study based on the omicron variant epidemic

**DOI:** 10.3389/fpubh.2025.1521658

**Published:** 2025-06-02

**Authors:** Mingwang Fu, Hualing Chen, Yongkang Qian, Yongjie Zhang, Haijian Guo, Ya Shen, Biyun Xu, Wantong Han, Haoran Zhou, Jinshui Xu, Bingwei Chen

**Affiliations:** ^1^Department of Epidemiology and Biostatistics, School of Public Health, Southeast University, Nanjing, China; ^2^Jiangsu Provincial Center for Disease Control and Prevention, Nanjing, China; ^3^Medical Statistics and Analysis Center, Nanjing Drum Tower Hospital, Nanjing University Medical School, Nanjing, China; ^4^Key Laboratory of Environmental Medicine Engineering, Ministry of Education, School of Public Health, Southeast University, Nanjing, China

**Keywords:** COVID-19, COVID-19 reinfection, SARS-CoV-2, primary healthcare workers, omicron variant

## Abstract

**Objectives:**

Since the global outbreak of SARS-CoV-2 in 2019, COVID-19 reinfection has become an increasing concern, particularly during the spread of the Omicron variant. Despite numerous international studies on COVID-19 reinfection, research focusing on healthcare workers, particularly those in primary care settings in mainland China, remains limited. This study aims to evaluate COVID-19 reinfection rates among primary healthcare workers (PHWs) in Jiangsu Province and to explore potential risk factors contributing to reinfection.

**Methods:**

This study utilized a combination of online questionnaires and on-site surveys to conduct two waves of investigation targeting PHWs after epidemic control policy adjustment in Jiangsu Province. Differences between the infection at the baseline visit and re-infection at the follow-up visit were analyzed, and multivariate logistic regression was used to assess the factors influencing reinfection.

**Results:**

A total of 5,541 PHWs were included in the study. At the baseline visit, the initial infection rate was 85.85% [95% confidence interval (CI): 84.93–86.77%], and the self-reported reinfection rate was 40.05% (95% CI: 38.65–41.44%). After adjustment, the reinfection rate was 29.41% (95% CI: 28.12–30.71%). The median reinfection interval between the two infections was 146 days (Interquartile range: 129–164 days). Logistic regression model revealed that female sex [odds ratio (OR) = 1.376, 95% CI: 1.190–1.592], history of fever clinic work (OR = 1.179, 95% CI: 1.045–1.330), working over 8 h per day (OR = 1.178, 95% CI: 1.040–1.336), being a nurse (OR = 1.201, 95% CI: 1.029–1.402), and a “less meat, more vegetables” diet (OR = 1.206, 95% CI: 1.020–1.426) were significant risk factors for reinfection. Additionally, regular physical exercise was found to be a protective factor (OR = 0.861, 95% CI: 0.754–0.983).

**Conclusion:**

COVID-19 reinfection rates were relatively high among PHWs in Jiangsu Province, particularly among women, nurses, those with fever clinic experience and working over 8 h per day. This study offers valuable insights for the prevention of COVID-19 reinfection and the development of protection strategies for PHWs. It is recommended that more targeted protective measures be implemented for high-risk groups, including appropriate work arrangements, regular health monitoring, and the promotion of healthy lifestyle habits.

## 1 Introduction

Since December 2019, SARS-CoV-2 has caused widespread and sustained global transmission. As of the seven days leading up to 15 September 2024, more than 776 million COVID-19 cases have been reported globally, with over 7.06 million deaths, according to estimates from the World Health Organization (1). However, the actual number of infections and deaths is likely much higher than reported (2). With the continuous mutation of SARS-CoV-2, the immune evasion capabilities of the virus have significantly increased, while human immunity wanes over time (3). This has resulted in frequent reports of reinfections, even multiple infections (4–8). However, research on reinfection among healthcare workers in mainland China, particularly those in primary care settings, remains relatively limited.

Since its first detection in November 2021, the Omicron variant has demonstrated a high degree of immune evasion. By 2022, Omicron had become the dominant variant in many countries worldwide (9). Numerous studies have shown that Omicron has a higher transmission rate and greater capacity to cause reinfections, with its reinfection rate surpassing that of earlier variants (10–12). Although Omicron appears to cause milder clinical manifestations compared to earlier variants, its significantly higher re-infection rate presents new challenges for public health control efforts (13, 14).

Unlike many other regions, mainland China successfully avoided large-scale nationwide outbreaks during the early stages of the pandemic through stringent control measures. However, following the adjustment of COVID-19 control policies to “normalized management” in December 2022, China experienced a surge in COVID-19 cases, reaching its first peak by the end of that month (15). Between September 26, 2022, and July 31, 2023, all 80,531 reported domestic COVID-19 cases in mainland China were caused by the Omicron variant (16). Due to their close contact with COVID-19 patients, primary healthcare workers (PHWs) faced a significantly higher risk of infection compared to the general population (17). Studies also indicate that repeated infections may result in more severe symptoms, with the risk of severe outcomes potentially increasing with each successive infection. In the context of ongoing SARS-CoV-2 mutations and adjustments to pandemic control policies, it is crucial to track infection and reinfection risks among healthcare workers. Effective preventive measures are needed to mitigate the risk of reinfection among this vulnerable population (18, 19).

This study investigates the infection and reinfection patterns among PHWs in Jiangsu Province during two distinct infection peaks. It aims to assess the characteristics and differences between the infection at baseline visit and re-infection at follow-up visit, and seeks to identify factors that may increase the risk of reinfection.

## 2 Materials and methods

### 2.1 Study design

This study employed a combination of online questionnaires and on-site surveys to conduct two waves of investigation among PHWs in Jiangsu Province. The first wave of the survey, conducted from January 17 to February 2, 2023, involved 34,090 individuals and took place approximately 6 weeks after the adjustment of COVID-19 prevention policies. The second wave of on-site survey, conducted from July 4 to July 20, 2023, targeted 5,754 PHWs from five counties included in the first wave. Professional staff collected data on infections occurring between April 1, 2023, and the survey date. All data were collected through the Questionnaire Star platform.^1^

The study protocol was approved by the Ethics Committee of the Jiangsu Provincial Center for Disease Control and Prevention (JSJK2023-B010-01). All participants voluntarily participated in the study and provided informed consent before completing the questionnaire.

### 2.2 Definitions

Some variables used in this study were defined as follows:

Individuals who self-reported infection: Defined as those meeting any of the following criteria: (1) positive nucleic acid test for SARS-CoV-2; (2) positive antigen test; (3) both nucleic acid and antigen tests positive; (4) exhibiting COVID-19-related symptoms without undergoing nucleic acid or antigen testing.

Uninfected individuals: Defined as those meeting any of the following criteria: (1) negative nucleic acid and/or antigen test; (2) the absence of COVID-19-related symptoms and no nucleic acid or antigen testing performed.

Individuals who self-reported an infection at baseline visit: Defined as those self-reported being infected with SARS-CoV-2 during the first wave of the survey.

Individuals who self-reported reinfection: Defined as those self-reported being infected during both waves of the survey.

Non-reinfected individuals: Defined as those infected during the first survey period but not confirmed to have been infected during the second survey period.

Reinfection interval (RI): The time interval between the self-reported infection at baseline visit and self-reported re-infection at the follow-up visit.

Body Mass Index (BMI): BMI was calculated by dividing weight (kg) by the square of height (m^2^). According to classification standards (20), BMI was categorized as follows: underweight (<18.5 kg/m^2^), normal weight (18.5–23.9 kg/m^2^), overweight (24.0–27.9 kg/m^2^), and obesity (≥28.0 kg/m^2^).

### 2.3 Survey questionnaire

The questionnaire used in this study was adapted from the second-round COVID-19 pneumonia questionnaire issued by the Chinese Center for Disease Control and Prevention and the Peking Union Medical College, and modified with reference to the “Diagnosis and Treatment Protocol for COVID-19 (Trial Version 10)” (21). The information collected by the questionnaire included: (1) Basic information: Demographic characteristics, medical history, occupation, lifestyle behaviors, and habits; (2) Infection-related information: Infection dates, typical symptoms (e.g., fever, muscle aches, cough, sore throat, nasal congestion, runny nose), hospitalization status, and any newly diagnosed diseases following infection; (3) Vaccination status: Number of vaccine doses and vaccine type; (4) Work burden: Weekly working hours and whether the participant had worked in fever clinics.

### 2.4 Statistical analysis

The sample size calculations resulted in *n* = 4,489, based on a projected long COVID prevalence of 8.89% and a two-sided 95% Clopper-Pearson confidence interval (CI) with a margin of error of 0.01778 (22). Considering that the reinfection rate exceeds the prevalence of long COVID, this sample size is deemed sufficient.

Continuous variables were expressed as means (standard deviation, SD), while categorical variables were summarized as frequencies and percentages. The 95% CI for the reinfection rate was calculated using the Wald method. For some individuals who exhibited COVID-19 symptoms but lacked nucleic acid tests or antigen detection, the adjusted reinfection rate was estimated using a weighted approach based on data from China’s National Influenza Surveillance System (16).

Statistical differences between groups were assessed by two-sample t-tests for continuous variables. For categorical variables, Pearson’s chi-square test was applied when the expected frequency in each cell was at least 5; otherwise, Fisher’s exact test was used. For the comparison of 14 symptom rates, the Bonferroni-Hochberg method was used for multiple comparisons. Both univariate and multivariate logistic regression analyses were conducted to identify factors associated with self-reported reinfection among PHWs. All variables were included in a multivariate logistic regression analysis using a stepwise selection method, with an entry criterion of *p* < 0.05 and a removal criterion of *p* > 0.10. Odds ratio (OR) and 95%CI quantified the risk associated with reinfection. A two-sided *p*-value < 0.05 was considered statistically significant.

Considering that some self-reported infected individuals had not undergone virus testing, resulting in potential ambiguity in the definitions, a sensitivity analysis was conducted. Nucleic acid or antigen-positive individuals were regarded as confirmed cases, while nucleic acid/antigen-negative or untested asymptomatic individuals were categorized as uninfected. Logistic regression was performed again using these groups.

All statistical analyses were performed using R software (version 4.3.3).

## 3 Results

### 3.1 Participants and self-reported reinfection rates

In the first wave of survey, a total of 34,090 questionnaires were collected between January 17 and February 2, 2023 in Jiangsu Province, the overall infection rate among PHWs was 81.05% (95% CI: 80.61–81.48%) from December 2022 to January 2023 (23). From these, five counties/districts were selected for second wave of on-site survey: Ganyu District in Lianyungang City, Funing County, Yandu District in Yancheng City, and the county-level cities of Kunshan and Changshu in Suzhou.

The second wave of the on-site survey included 5,754 PHWs across five counties. After data verification, questionnaires with inconsistent or illogical responses were excluded, resulting in the removal of 213 questionnaires. A total of 5,541 valid questionnaires remained for analysis.

Among the 5,541 participants included in this study, 67.30% were female, with a mean age of 39.80 years (SD = 10.80). Of the 4,757 participants who self-reported an infection at baseline visit, 1,905 were self-reported reinfected, and 2,852 did not experience reinfection. The self-reported infection at baseline visit rate was 85.85% (95% CI: 84.93–86.77%), and the self-reported reinfection rate was 40.05% (95% CI: 38.65–41.44%). Among those reporting an infection at baseline visit, 6.71% exhibited COVID-19-related symptoms without having undergone nucleic acid or antigen testing. In the second wave survey, 1,253 participants had a positive nucleic acid test, positive antigen test, or both, while 652 participants (34.23%) had COVID-19-related symptoms without testing. Based on the positivity rate among influenza-like illness patients from national sentinel hospitals, we assumed that a proportion of these 652 symptomatic participants were false positives. By adding the confirmed positives to the numerator and incorporating the estimated number of false positives into the denominator, we recalculated the positivity rate, yielding an adjusted reinfection rate of 29.41% (95% CI: 28.12–30.71%).

### 3.2 Analysis of infection at the baseline visit and follow-up visit

#### 3.2.1 Infection dates and time intervals

**Figure 1:**
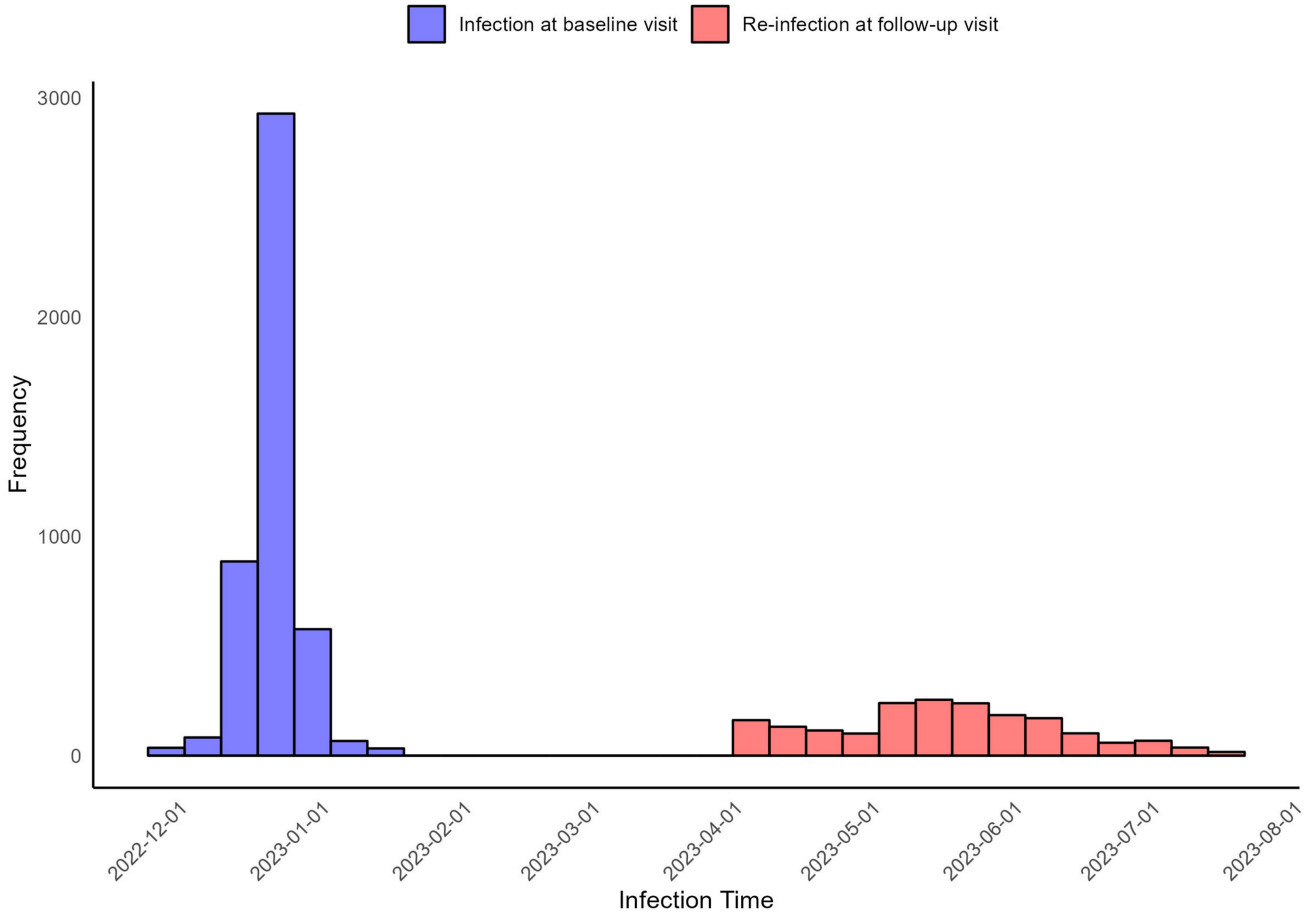
Distribution of infection dates.

**Figure 2:**
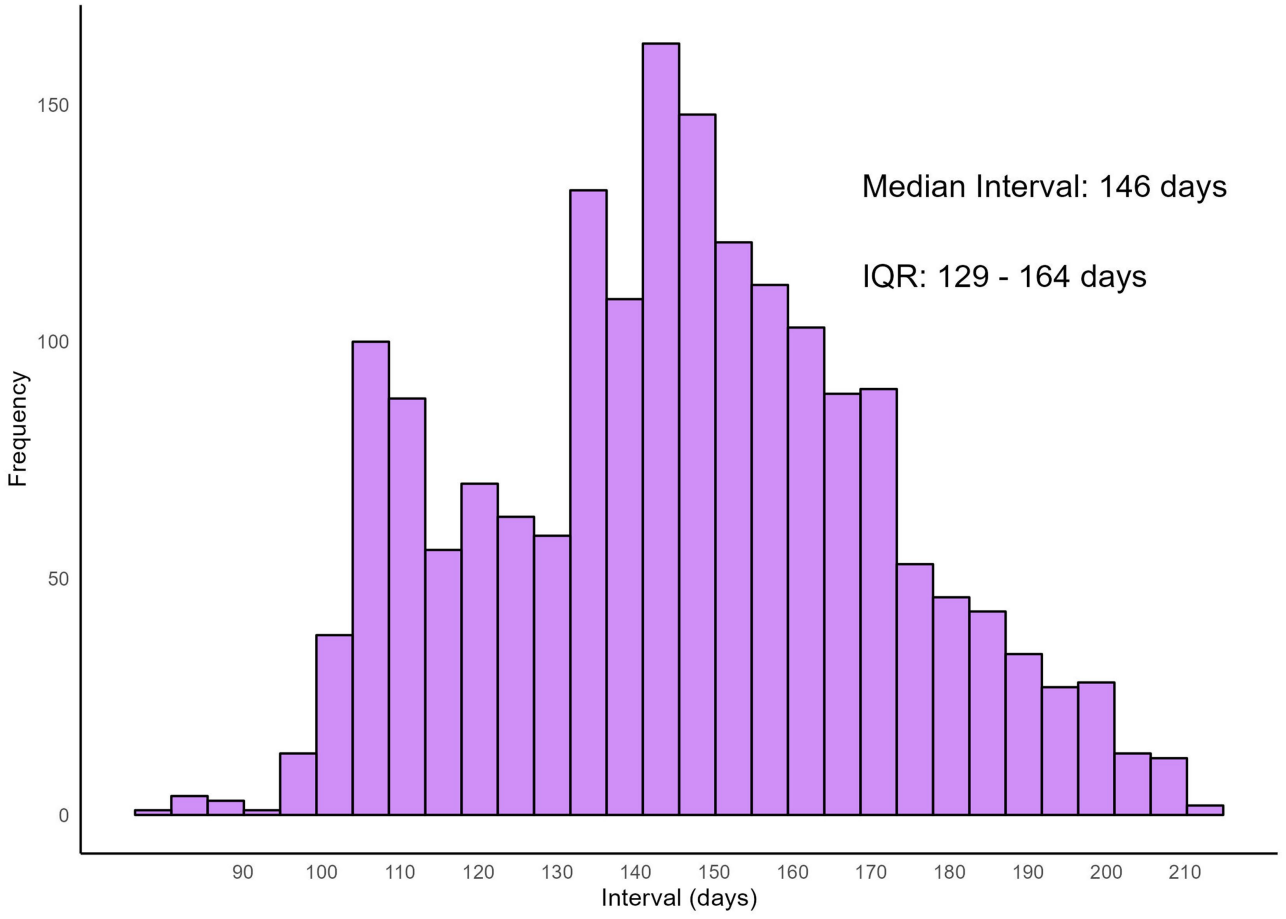
Distribution of reinfection intervals.

The distribution of infection dates for both self-reported infections at baseline visit and reinfections is illustrated using histograms (Figure 1). Infections at baseline visit were concentrated in late December 2022, while reinfections exhibited a more dispersed pattern, peaking in May 2023, followed by a gradual decline in infection numbers. Figure 2 presents reinfection intervals (RIs), the median RI was 146 days [Interquartile Range (IQR): 129–164 days], with the shortest and longest RIs being 77 days and 211 days, respectively.

#### 3.2.2 Newly diagnosed diseases and symptom comparison between infection at baseline visit and re-infection at follow-up visit

**Figure 3:**
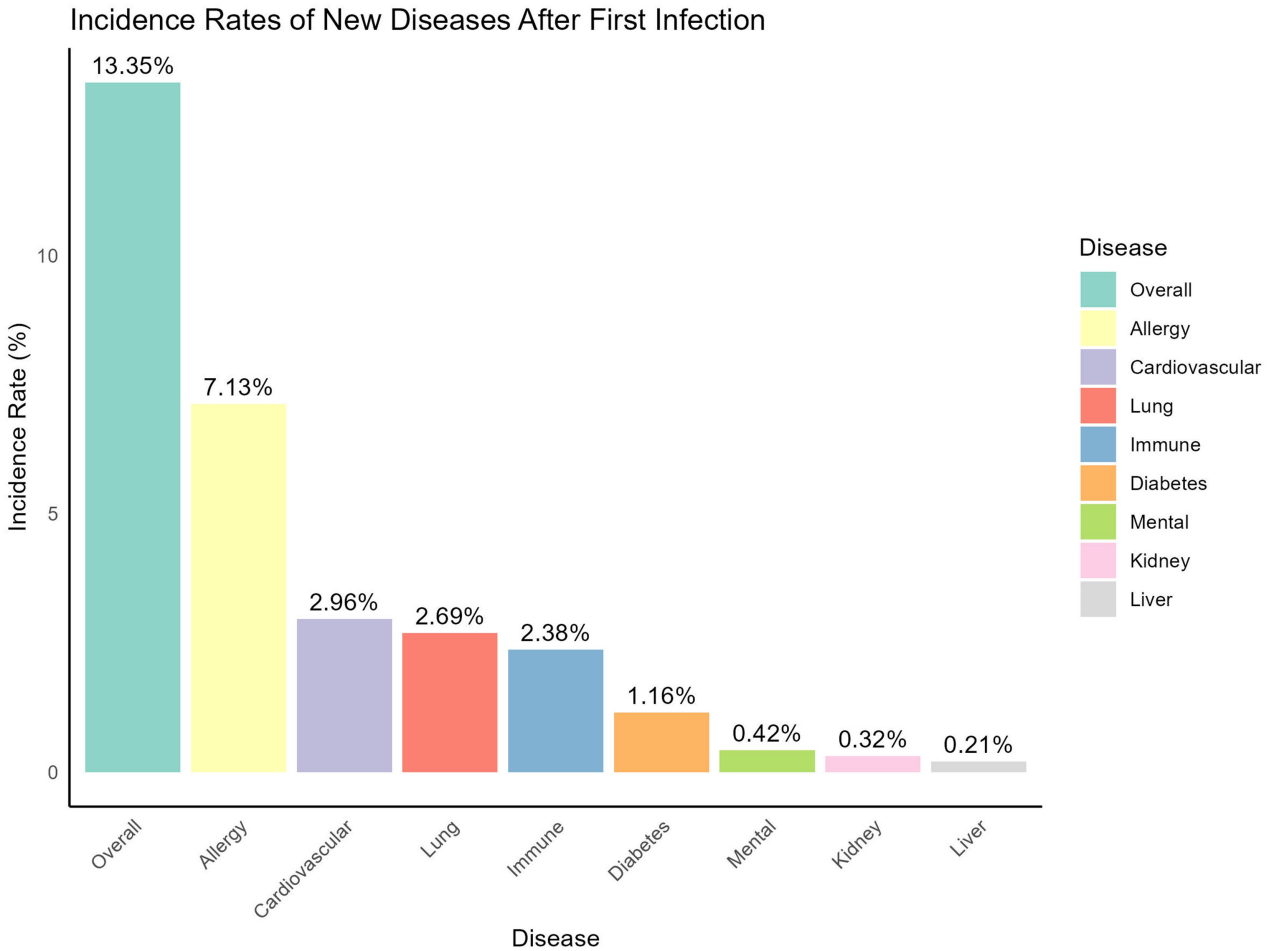
Newly diagnosed diseases after infection at baseline visit.

As shown in Figure 3, 13.35% of participants who experienced an infection at baseline visit reported developing one or more newly diagnosed diseases afterward. The most frequently reported condition was allergic diseases, such as asthma and rhinitis, affecting 7.13% of participants. This was followed by cardiovascular disease (2.96%), lung disease (2.69%), immune system disorders (2.38%), diabetes (1.16%), mental health issues (0.42%), kidney disease (0.32%), and liver disease (0.21%).

**Figure 4:**
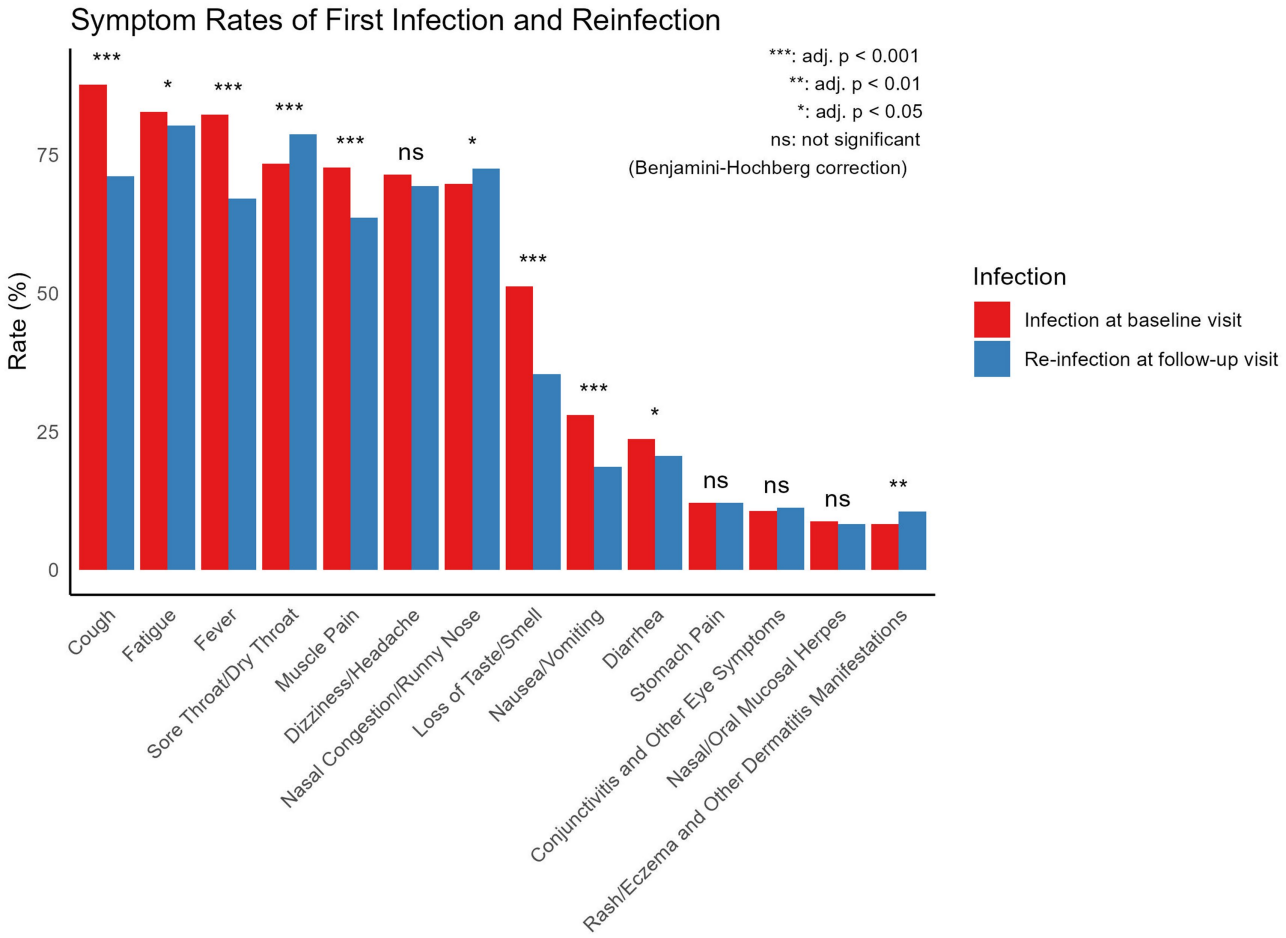
Comparison of symptom frequencies between infection at baseline visit and self-reported re-infection at follow-up visit. All symptom comparisons were performed using Chi-square tests.

When comparing symptom frequencies between the infection at baseline visit and re-infection at Follow-up Visit (Figure 4), the main symptoms during the infection at baseline visit were cough (87.81%), fatigue (82.83%), fever (82.38%), sore throat/dry throat (73.45%), muscle pain (72.73%), and nasal congestion/runny nose (69.77%). During the reinfection, the main symptoms included fatigue (80.37%), sore throat/dry throat (78.74%), nasal congestion/runny nose (72.60%), cough (71.23%), dizziness/headache (69.45%), and fever (67.14%). In comparison, cough, diarrhea, fatigue, fever, loss of taste/smell, muscle pain, and nausea/vomiting were significantly more frequent during the infection at baseline visit, while nasal congestion/runny nose, rash, eczema and other dermatological manifestations, and sore throat/dry throat were significantly more frequent during the reinfection. All comparisons among the symptoms were performed using chi-square tests.

#### 3.2.3 Severity of infections

**Figure 5:**
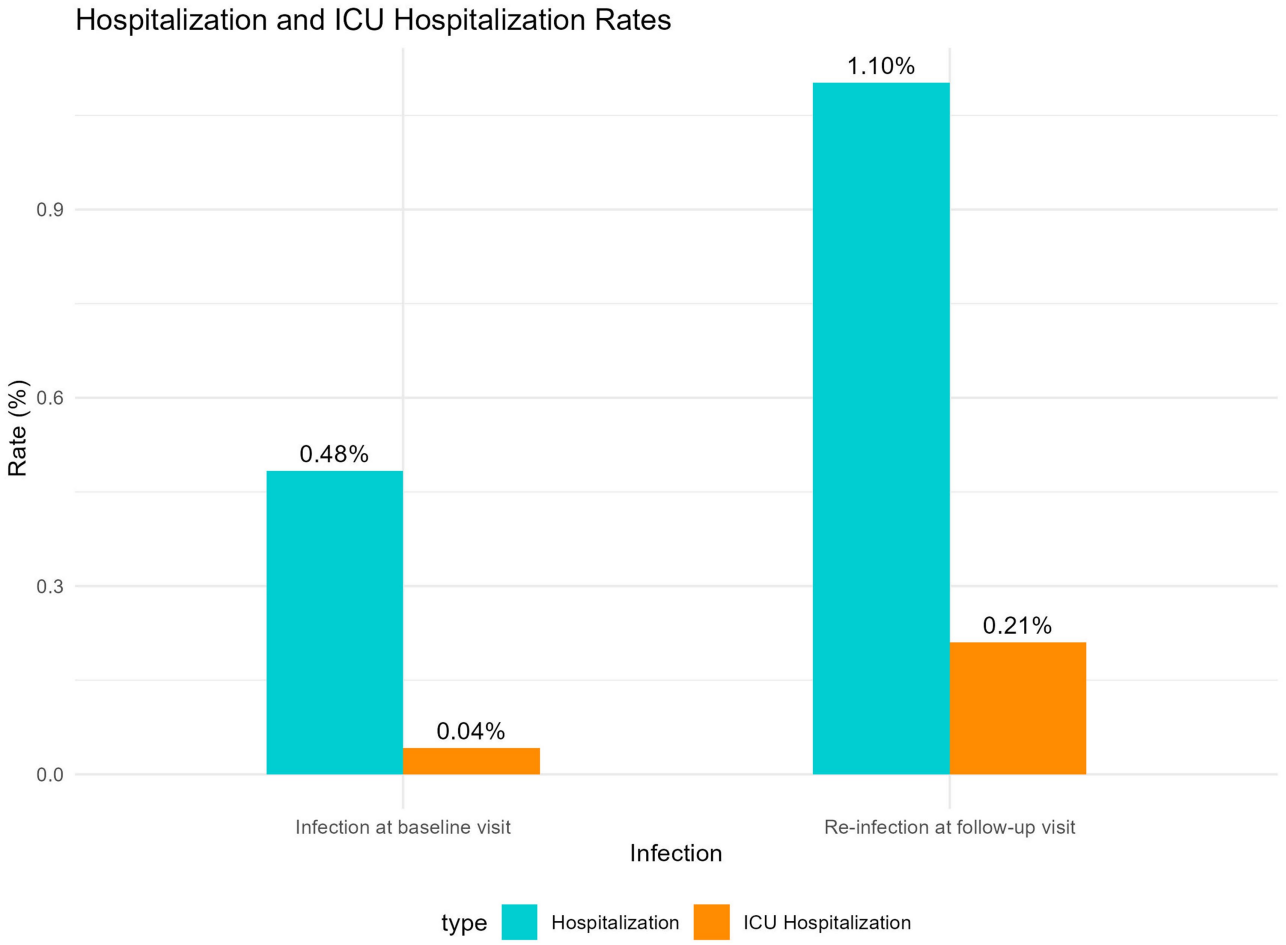
Comparison of hospitalization and ICU admission rates between infection at baseline visit and re-infection at follow-up visit.

Among self-reported reinfected participants, 73.65% reported that their symptoms during the infection at baseline visit were more severe, 14.5% indicated that the severity of symptoms were similar between the two waves, and only 11.9% perceived that their symptoms were more severe during the reinfection. However, the hospitalization rate (1.10%) during reinfection was significantly higher compared to the hospitalization rate (0.48%) during the infection at baseline visit (*p* < 0.05, Chi-squared test). The ICU admission rates during the follow-up visit and at the baseline visit were 0.21 and 0.04%, respectively. The difference between the two groups was not statistically significant (*p* = 0.059, Fisher’s exact test), as shown in Figure 5.

### 3.3 Comparison between reinfected and non-reinfected groups

#### 3.3.1 Univariate analysis

Univariate analysis was performed to compare the baseline characteristics of 1,905 reinfected individuals and 2,852 non-reinfected individuals, as shown in Table 1. Females had a significantly higher reinfection rate (42.74%) compared to males (34.10%), indicating that females may face a higher risk of reinfection. In addition, nurses had the highest reinfection rate, followed by medical technicians and pharmacists. Regarding vaccination status, those who had received 0–1 doses of the vaccine had the highest reinfection rate (47.66%). The reinfection rate decreased as the number of vaccine doses increased, and the differences between the various dose groups were not statistically significant. Chi-square test was used in all univariate analyses, statistically significant differences were observed among the variables gender, smoking, drinking, weekly exercise, history of fever clinic, diet structure and position.

**Table 1:** Univariate analysis for factors associated with Self-reported reinfection.

Variables	Non-reinfected group	Reinfected group	Total	*P*-value
Age (mean ± SD)	39.71 ± 10.98	39.30 ± 10.02	39.55 ± 10.61	0.175
Gender				<0.001
Female	1,874(57.26%)	1,399(42.74%)	3,273	
Male	978(65.9%)	506(34.1%)	1,484	
BMI				0.695
Normal weight	1,468 (59.63%)	994 (40.37%)	2,462	
Obesity	348 (58.68%)	245 (41.32%)	593	
Overweight	882 (61.17%)	560 (38.83%)	1,442	
Underweight	154 (59.23%)	106 (40.77%)	260	
Smoking				0.006
No	2,576 (59.34%)	1,765 (40.66%)	4,341	
Yes	276 (66.35%)	140 (33.65%)	416	
Drinking				<0.001
No	2,358 (58.88%)	1,647 (41.12%)	4,005	
Yes	494 (65.69%)	258 (34.31%)	752	
Weekly exercise				0.004
No	746 (56.64%)	571 (43.36%)	1,317	
Yes	2,106 (61.22%)	1,334 (38.78%)	3,440	
Management				0.573
No	2,375 (59.76%)	1,599 (40.24%)	3,974	
Yes	477 (60.92%)	306 (39.08%)	783	
Daily working hours				0.122
≤8 h	1,850 (60.80%)	1,193 (39.20%)	3,043	
>8 h	1,002 (58.46%)	712 (41.54%)	1,714	
History of fever clinic				0.004
No	1,492 (61.99%)	915 (38.01%)	2,407	
Yes	1,360 (57.87%)	990 (42.13%)	2,350	
Diet structure				0.049
Balanced	2,100 (60.90%)	1,348 (39.10%)	3,448	
Less meat, more veg.	381 (55.95%)	300 (44.05%)	681	
More meat, less veg.	371 (59.08%)	257 (40.92%)	628	
Diabetic				0.380
No	2,778(59.84%)	1,864(40.16%)	4,642	
Yes	74(64.35%)	41(35.65%)	115	
History of cardiovascular disease				0.043
No	2,645(59.56%)	1,796(40.44%)	4,441	
Yes	207(65.51%)	109(34.49%)	316	
Vaccine doses				0.206
0 ~ 1	56(52.34%)	51(47.66%)	107	
2 ~ 3	1,520(60.61%)	988(39.39%)	2,508	
≥4	1,276(59.57%)	866(40.43%)	2,142	
Position				<0.001
Doctor	1,318(62.23%)	800(37.77%)	2,118	
Medical technician	333(59.57%)	226(40.43%)	559	
Nurse	733(54.74%)	606(45.26%)	1,339	
Other position	271(65.46%)	143(34.54%)	414	
Pharmacist	197(60.24%)	130(39.76%)	327	

#### 3.3.2 Multivariate logistic regression model

The final model (Table 2) indicated that female gender (OR = 1.376, 95% CI: 1.190–1.592), history of fever clinic work (OR = 1.179, 95% CI: 1.045–1.330), working over 8 h per day (OR = 1.178, 95% CI: 1.040–1.336), and a “less meat, more vegetables” diet (OR = 1.206, 95% CI: 1.020–1.426), along with being a nurse (OR = 1.201, 95% CI: 1.029–1.402), were significant risk factors for reinfection. Regular weekly exercise was identified as a protective factor (OR = 0.861, 95% CI: 0.754–0.983).

**Table 2:** Factors associated with Self-reported reinfection in univariate and multivariate logistic regression.

Variable	Univariate analysis		Multivariate analysis	
	OR (95% CI)	*P*-value	OR (95% CI)	*P*-value
Age	0.996 (0.991–1.002)	0.182		
Gender				
Male	1.000		1.000	
Female	1.443 (1.270–1.639)	<0.001	1.376 (1.190–1.592)	<0.001
BMI				
Normal weight	1.000			
Overweight	0.938 (0.821–1.071)	0.343		
Obesity	1.04 (0.867–1.248)	0.675		
Under weight	1.017 (0.784–1.319)	0.902		
Weekly exercise	0.828 (0.728–0.941)	0.004	0.861 (0.754–0.983)	0.027
History of fever clinic work	1.187 (1.057–1.333)	0.004	1.179 (1.045–1.330)	0.007
Daily working hours				
≤8 h	1.000		1.000	
>8 h	1.102 (0.977–1.243)	0.115	1.178 (1.040–1.336)	0.010
Diet structure				
Balanced			1.000	
More meat, less veg.	1.079 (0.908–1.283)	0.388	1.09 (0.914–1.301)	0.337
Less meat, more veg.	1.227 (1.039–1.448)	0.016	1.206 (1.020–1.426)	0.028
Position				
Doctor	1.000		1.000	
Medical technician	1.118 (0.924–1.353)	0.25	1.149 (0.946–1.396)	0.160
Nurse	1.362 (1.185–1.565)	<0.001	1.201 (1.029–1.402)	0.021
Pharmacist	1.087 (0.857–1.380)	0.492	1.041 (0.816–1.328)	0.749
Other position	0.869 (0.697–1.084)	0.214	0.892 (0.711–1.118)	0.322
Vaccine doses				
0 ~ 1	1.000		1.000	
2 ~ 3	0.714 (0.484–1.052)	0.088	0.718 (0.486–1.062)	0.097
≥4	0.745 (0.505–1.100)	0.138	0.795 (0.537–1.178)	0.253
Management	0.953 (0.814–1.115)	0.546		
Smoking	0.74 (0.599–0.915)	0.005		
Drinking	0.748 (0.635–0.880)	<0.001		
Cardiovascular disease	0.775 (0.610–0.985)	0.038		
Diabetic	0.826 (0.561–1.215)	0.331		

OR, odds ratio; CI, confidence interval; Veg., vegetables.

#### 3.3.3 Sensitivity analysis

The sensitivity analysis results (Table 3) were highly consistent with the main analysis, with female gender (OR = 1.413, 95% CI: 1.190–1.679), a “less meat, more vegetables” diet (OR = 1.256, 95% CI: 1.031–1.529), fever clinic work (OR = 1.255, 95% CI: 1.089–1.447), and being a nurse (OR = 1.278, 95% CI: 1.067–1.530) remaining significant risk factors for reinfection. Medical technician (OR = 1.319, 95% CI: 1.051–1.657) was identified as a risk factor only in the sensitivity analysis. Vaccine doses of 2 ~ 3 was identified as a protective factor (OR = 0.630, 95% CI: 0.406–0.977). These findings demonstrate that regardless of the infection definition used, certain risk factors consistently contribute to reinfection, further confirming the robustness of the results.

**Table 3:** Sensitivity analysis logistic regression results.

Variable	Univariate analysis		Multivariate analysis	
	OR (95% CI)	*P*-value	OR (95% CI)	*P*-value
Age	0.998 (0.992–1.004)	0.542		
Gender				
Male	1.000		1.000	
Female	1.478 (1.269–1.722)	<0.001	1.413 (1.190–1.679)	<0.001
BMI				
Normal weight	1.000			
Overweight	0.984 (0.842–1.151)	0.845		
Obesity	1.009 (0.814–1.252)	0.932		
Under weight	1.031 (0.758–1.401)	0.847		
Weekly exercise	0.883 (0.758–1.029)	0.110		
History of fever clinic work	1.241 (1.082–1.423)	0.002	1.255 (1.089–1.447)	0.002
Daily working hours				
≤8 h	1.000		1.000	
>8 h	1.072 (0.930–1.236)	0.338	1.144 (0.988–1.326)	0.073
Diet structure				
Balanced			1.000	
More meat, less veg.	1.085 (0.886–1.329)	0.431	1.120 (0.912–1.375)	0.279
Less meat, more veg.	1.276 (1.050–1.550)	0.014	1.256 (1.031–1.529)	0.023
Position				
Doctor	1.000		1.000	
Medical technician	1.259 (1.009–1.573)	0.042	1.319 (1.051–1.657)	0.017
Nurse	1.444 (1.228–1.698)	<0.001	1.278 (1.067–1.530)	0.008
Pharmacist	1.03 (0.774–1.371)	0.838	1.004 (0.750–1.345)	0.977
Other position	0.854 (0.653–1.116)	0.248	0.891 (0.678–1.172)	0.411
Vaccine doses				
0 ~ 1	1.000		1.000	
2 ~ 3	0.632 (0.410–0.976)	0.038	0.630 (0.406–0.977)	0.039
≥4	0.693 (0.449–1.070)	0.098	0.727 (0.469–1.129)	0.156
Management	0.92 (0.764–1.108)	0.38		
Smoking	0.691 (0.533–0.897)	0.005		
Drinking	0.738 (0.607–0.898)	0.002		
Cardiovascular disease	0.792 (0.597–1.051)	0.106		
Diabetic	1.048 (0.665–1.652)	0.840		

OR, odds ratio; CI, confidence interval; Veg., vegetables.

## 4 Discussion

In this study, we analyzed the reinfection characteristics and potential risk factors among PHWs during the Omicron variant wave. Female PHWs, nurses, and individuals with a history of fever clinic work were found to have a significantly higher risk of re-infection. Additionally, working more than 8 h per day and having a “less meat, more vegetables” diet were identified as risk factors, while regular weekly exercise was a protective factor. Vaccine doses of 2 ~ 3 was identified as a protective factor against reinfection in the sensitivity analysis. These findings highlight important occupational and behavioral factors associated with COVID-19 reinfection, underscoring the need for targeted protective measures for high-risk healthcare workers.

The results indicate that after adjustments to epidemic control policies, the two major waves of Omicron infections in Jiangsu occurred at the end of 2022 and mid-2023, with a median reinfection interval of 146 days (IQR: 129–164 days). Moreover, first-time COVID-19 infections may be associated with the onset of various diseases, particularly allergic conditions (e.g., asthma, rhinitis), with 7.13% of patients reporting new allergic conditions after infection. Although these comorbidities may not be directly caused by COVID-19—such as the development of diabetes potentially due to natural aging—the higher rates of allergies, immune system, pulmonary, and cardiovascular conditions suggest that COVID-19 could have a broader impact on these systems, providing a direction for future research into long-COVID effects (24, 25). The study also observed that primary infections tended to present more systemic symptoms (e.g., fever, cough, fatigue), while reinfections predominantly involved upper respiratory symptoms (e.g., sore throat, nasal congestion). This pattern may reflect the immune system’s response to reinfection. During the first infection, the immune system is in its initial response phase, resulting in a stronger systemic reaction.

In terms of reinfection rates, the study found that the primary infection rate among frontline healthcare workers in Jiangsu was 85.85% (95% CI: 84.93–86.77%), and the reinfection rate was 40.05% (95% CI: 38.65–41.44%). After adjusting for sentinel hospital influenza-like illness SARS-CoV-2 positivity rates, the recalculated reinfection rate was 29.41% (95% CI: 28.12–30.71%). Flacco et al.’s meta-analysis from June 2022 found that reinfection rates before the Omicron wave were 0.97%, rising to 3.31% in the first 3 months of Omicron’s spread (26). Similarly, research by Fonseca et al. in Brazil found a reinfection rate of up to 8%, which increased over time (27). Wei et al., utilizing data from the UK Office for National Statistics (ONS) COVID-19 Infection Survey, reported that reinfection rates rose from 10–11% to 14–16% (28). In China, a retrospective study by Zhang et al. in Shanxi Province found that the reinfection rate could reach as high as 25.1% (29). Additionally, research by Cai et al. in Guangdong Province indicated a reinfection rate of 18.4% following an initial Omicron infection, with an overall reinfection rate of 28.3% (30). The reinfection rates observed in these studies are consistent with our findings on the Chinese population. The relatively high reinfection rates in our study may be attributed to concentrated infection clusters during the adjustment of national control policies, potentially resulting in a higher reinfection rate compared to other countries.

The severity of reinfection remains a contentious issue, with no clear consensus in the literature (31). Some studies suggest that reinfection leads to milder symptoms, with lower hospitalization and mortality rates (32). However, other studies report an increased risk of hospitalization and death during reinfection, with cumulative risks and burdens rising with each subsequent infection (33). Nguyen et al. found no significant difference in symptom severity between primary and secondary infections (19). In our study, most participants perceived their reinfection symptoms as milder than their initial infection, while the hospitalization and ICU admission rates for reinfection were significantly higher. This discrepancy may be related to strained healthcare resources during the first wave of infections, such as shortages of hospital bed, which led to lower hospitalization rates during primary infections. Hadley et al. found a statistically significant association between the severity of the first and subsequent infections, with individuals experiencing severe first infections showing a higher risk of hospitalization and death upon reinfection (34). These findings underscore the importance of studying the severity of COVID-19 reinfection, which is crucial for assessing disease burden and shaping future public health policies.

The multivariate logistic regression analysis indicated that being female, having a history of working in fever clinics, and being a nurse were significant risk factors for reinfection. The higher reinfection risks for females and nurses might be related to their higher occupational exposure frequency, particularly nurses who often work in high-risk environments on the frontlines of care. Furthermore, experience working in fever clinics increases exposure risks, highlighting the need for strengthened occupational protections for healthcare workers, particularly for high-risk groups. Only in the sensitivity analysis, Vaccine doses of 2~3 was identified as a protective factor against reinfection. Although the OR values for both 2~3 and ≥4 doses were less than 1.000, most of the *p*-values were greater than 0.05. This may be due to the small number of individuals with Vaccine doses of 0~1, as only 2.25% of PHWs received one or fewer doses. This may also suggest that maintaining high immunity levels through vaccine boosters is critical in the context of ongoing SARS-CoV-2 mutations. Future vaccination strategies should consider the waning efficacy of vaccines over time, and regular booster doses may be an effective way to reduce reinfection risks (35).

Increasing the supply of personal protective equipment, rationalizing work hours, implementing regular health monitoring, and prioritizing vaccination for these groups could effectively reduce infection risks.

However, this study has several limitations. First, data collection involved a combination of online and offline methods, with online questionnaires used during the first wave and on-site during the second. This discrepancy may have led to inconsistencies in the accuracy of responses. Second, due to the extensive scale of the outbreak, it was not feasible to test all participants for SARS-CoV-2 using nucleic acid or antigen tests. Consequently, individuals who were not tested but exhibited symptoms were classified as infected, which may have overestimated infection and reinfection rates. To mitigate these limitations, we adjusted the reinfection rate using data from sentinel hospitals, and conducted a sensitivity analysis to validate the robustness of the results. However, some residual errors may still remain.

In conclusion, this study identified higher reinfection risks for female healthcare workers, nurses, “less meat, more vegetables” diet and those with fever clinic experience. It also highlighted significant differences in the symptoms and severity of primary and secondary infections. These findings provide important insights into the risk factors for reinfection and strategies for its prevention.

## 5 Conclusion

This study examined the characteristics of COVID-19 primary infections and reinfections among PHWs during the Omicron wave in Jiangsu Province and identified potential risk factors for reinfection. The results showed a primary infection rate of 85.85% and a reinfection rate of 40.05%, with an adjusted reinfection rate of 29.41%. The study also highlighted differences in symptomatology and severity between primary infections and reinfections. While primary infections were more systemic in nature, reinfections predominantly involved upper respiratory symptoms. Although most participants perceived their reinfection symptoms as milder, the hospitalization and ICU admission rates were significantly higher during reinfection compared to primary infections, possibly due to healthcare resource constraints during the first infection wave.

Female healthcare workers, nurses, and individuals with a history of working in fever clinics were found to have a significantly higher risk of reinfection. Additionally, working more than 8 h per day and following a “less meat, more vegetables” diet were associated with increased reinfection risk, while regular weekly exercise was identified as a protective factor. These findings underscore the need for targeted protective measures for high-risk healthcare workers, particularly females, nurses, and those with fever clinic experience. Enhancing personal protective equipment supplies, rationalizing work hours, and implementing regular health monitoring for these high-risk groups may effectively reduce the risk of reinfection. By improving workplace protections and health measures for healthcare workers, the risk of reinfection can be mitigated, further safeguarding this critical population.

## Data Availability

The raw data supporting the conclusions of this article will be made available by the authors, without undue reservation.

## Footnotes

^1^ https://www.wjx.cn/
